# Fatal Infection in a Wild Sandbar Shark (*Carcharhinus plumbeus*), Caused by *Streptococcus agalactiae*, Type Ia-ST7

**DOI:** 10.3390/ani10020284

**Published:** 2020-02-12

**Authors:** Danny Morick, Nadav Davidovich, Eyal Bigal, Ezra Rosenbluth, Arieli Bouznach, Assaf Rokney, Merav Ron, Natascha Wosnick, Dan Tchernov, Aviad P. Scheinin

**Affiliations:** 1 Department of Marine Biology, Leon H. Charney School of Marine Sciences, University of Haifa, Haifa 3498838, Israel; eyalbigal@gmail.com (E.B.); dtchernov@univ.haifa.ac.il (D.T.); shani.aviad@gmail.com (A.P.S.); 2 Morris Kahn Marine Research Station, University of Haifa, Haifa 3498838, Israel; 3 Israeli Veterinary Services, Bet Dagan 5025001, Israel; Nadavd@moag.gov.il; 4 Kimron Veterinary Institute, Bet Dagan 5025001, Israel; ezrar@moag.gov.il (E.R.); Arielib@moag.gov.il (A.B.); 5 Government Central Laboratories, Ministry of Health, Jerusalem 91342, Israel; assaf.rokney@moh.gov.il (A.R.); merav.ron@moh.gov.il (M.R.); 6 Departamento de Fisiologia, Centro Politécnico, Universidade Federal do Paraná, Curitiba 80060-000, Brazil; n.wosnick@gmail.com

**Keywords:** *Carcharhinus plumbeus*, sandbar shark, *Streptococcus agalactiae*, streptococcosis, type Ia-ST7, whole genome sequencing (WGS), phylogeny

## Abstract

**Simple Summary:**

*Streptococcus agalactiae* (group B *Streptococcus*, GBS) is a major fish pathogenic bacterium. In this study, we describe a fatal infection of a stranded wild sandbar shark (*Carcharhinus plumbeus*) by a post-mortem examination, histopathology, classical bacteriology and advanced molecular methods. The bacterial agent was characterized as *S. agalactiae*, type Ia-ST7.

**Abstract:**

*Streptococcus agalactiae* is one of the most important fish pathogenic bacteria as it is responsible for epizootic mortalities in both wild and farmed species. *S. agalactiae* is also known as a zoonotic agent. In July 2018, a stranded wild sandbar shark (*Carcharhinus plumbeus*), one of the most common shark species in the Mediterranean Sea, was found moribund on the seashore next to Netanya, Israel, and died a few hours later. A post-mortem examination, histopathology, classical bacteriology and advanced molecular techniques revealed a bacterial infection caused by *S. agalactiae*, type Ia-ST7. Available sequences publicly accessible databases and phylogenetic analysis suggest that the *S. agalactiae* isolated in this case is closely related to fish and human isolates. To the best of our knowledge, this is the first description of a fatal streptococcosis in sandbar sharks.

## 1. Introduction

The sandbar shark, *Carcharhinus plumbeus* (Nardo, 1827), is a wide-ranging coastal species found in tropical and temperate regions and is one of the most common shark species in the Mediterranean Sea [1]. Although it is a common species, information on its diet and feeding habits are scarce. Moreover, knowledge about its health status and the prevalence of diseases within the species is extremely limited.

Streptococcosis is a septicemic disease that affects freshwater, brackish and marine fish in both wild and farmed populations. *Streptococcus agalactiae* (group B *Streptococcus*, GBS) is an important fish-pathogenic bacterium, frequently associated with septicemia and meningoencephalitis in fish [2]. The first outbreak of streptococcosis in fish was recorded in 1958 and infected rainbow trout, *Oncorhynchus mykiss*, cultured in Japan [3]. It was later reported in different saltwater species, both in Osteichtyes (bony) and Chondrichthyes (cartilaginous) fish [4,5]. 

Publications describing wild fish infections caused by *S. agalactiae* are much less common in comparison to those describing infections in farmed fish populations [6]. For example, the *S. agalactiae*-infection was reported in wild mullet (*Liza klunzinger*) in Kuwait in 2002 [7]. Moreover, in 2012 *S. agalactiae* has also been described as causing deaths of different wild fish species, namely, adult Queensland grouper (*Epinephelus lanceolatus*), javelin grunter (*Pomadasys kaaken*), giant sea catfish (*Netuma thalassina*) and squaretail mullet (*Ellochelon vaigensis*) in north Queensland, Australia [8]. Another publication revealed an infection caused by *S. agalactiae* in wild and cultured Gulf Killifish (*Fundulus grandis*) from coastal waters in the Gulf of Mexico, USA (2015) [9].

The number of elasmobranchs streptococcosis cases are extremely limited. The first streptococcal infection in cartilaginous fish was reported in 1992. The authors communicated a Lancefield Group B *Streptococcus* septicemia that was diagnosed in a long-term captive female nurse shark (*Ginglymostoma cirratum*) in the USA [10]. The mortalities caused by *S. agalactiae* involving different species of wild and captive rays were described in Australia (2009–2010). These included mortalities of mangrove whiprays (*Himantura granulata*), estuary rays (*Dasyatis fluviorum*), eastern shovelnose rays (*Aptychotrema rostrata*), white-spotted eagle rays (*Aetobatus narinari*) and blue-spotted mask rays (*Neotrygon kuhlii*). Several *S. agalactiae*-serotyps were found based on the complete *cps* sequences, including a case of *S. agalactiae* Ia serotype (acc. no. NZ_AAJP00000000.1) [5].

*S. agalactiae* exhibits a wide host-range, including both homoeothermic and poikilothermic animals [11]. It is also a zoonotic agent that may cause morbidity in neonates and can cause septicemia and meningitis in immuno-compromised elderly people [12]. Since the first description of GBS in hatchery-reared freshwater fish in the USA in 1966 [13], reports of GBS in fish have increased. 

In July 2018, a female shark was observed moribund on Netanya's shoreline (Video S1, with permission from Ousama Hason) and died a few hours later. A post-mortem examination, histopathology, classical bacteriology and advanced molecular techniques were performed to identify the cause of death.

## 2. Materials and Methods

At the laboratory, the carcass was measured, a post-mortem examination was conducted and tissue samples were collected. Samples of liver, kidney, intestine, gonads, heart and brain were fixed in buffered formalin 10% for 48 h. Subsequently, the fixed samples were reduced in size, dehydrated and embedded in Paraplast^®^, as per the standard histological protocols. Three µm thin sections were stained alternatively with Mayer’s haematoxyline and eosine (H&E) and Ziehl–Neelsen stain (ZN), mounted in Eukitt^®^ resin and observed with a Leitz-Diaplan microscope at 40–1000 magnifications. Digital images were recorded by using an integrated Leica MC170HD (Leica, UK) camera and LAS 4.5.0 (Leica, UK) software. 

For bacteriological examination, tissue samples of liver, spleen, kidney, uterine fluid and brain were aseptically collected. Cultures were then performed on blood agar and tryptone soya agar (TSA) and incubated for 48–72 h at 25 °C. Bacterial colonies, referring to the genus *Streptococcus*, appeared on the agar plates 48 h post-planting. 

DNA was extracted from the colonies isolated from the brain tissue using a Wizard SV Genomic System (Promega, WI, USA) and the genomic DNA purification protocol following the manufacturer’s instructions for tissue lysates. The quantity and purity of the DNA were estimated using NanoDrop One (Thermo Scientific, Rockford, CA, USA). The genomic DNA obtained was stored at −20 °C until use.

Amplification and sequencing of the 16S rRNA region was performed by Hy-Labs (Rehovot, Israel). The sequence was edited using a Sequencher 4.0 (Gene Codes Corporation, Ann Arbor, MI, USA) and compared to sequences available on GenBank. The 16S rRNA sequence from this study was submitted to GenBank (acc. no. MK517599). 

The *S. agalactiae* isolate was then serotyped at the National Reference Laboratory (Ministry of Health, Israel) using a molecular serotyping method for epidemiological tracing. A multiplex-PCR for species confirmation and direct identification of *S. agalactiae* capsular type was performed [9]. Additionally, whole genome sequencing (WGS) was performed. DNA was extracted using the QIAsymphony^®^ SP system and the QIAsymphony^®^ DNA mini kit (Qiagen) according to the manufacturers’ recommendations. A DNA library was prepared using the Nextera XT library preparation kit (Illumina, CA, USA) followed by WGS using the Illumina MiSeq system with the read length of 250 bp paired-end. Reads were assembled using SPAdes by the BioNumerics 7.6.3 platform. The assembled genome was submitted to the PubMLST *S. agalactiae* database as ICLGBS001 (ST33917). An analysis of wgMLST was performed for ST-7 strains, which are publicly available in the PubMLST *S. agalactiae* database using the Genome Comparator (GC) tool. Allelic profilesof the 1,914 loci were retrieved from GC and imported to BioNumerics in order to generate a phylogenetic tree. In light of this, we compared the local *S*. *agalactiae* WGS with other ST-7 sequences available on pubMLST by wgMLST.

## 3. Results

**Figure 1:**
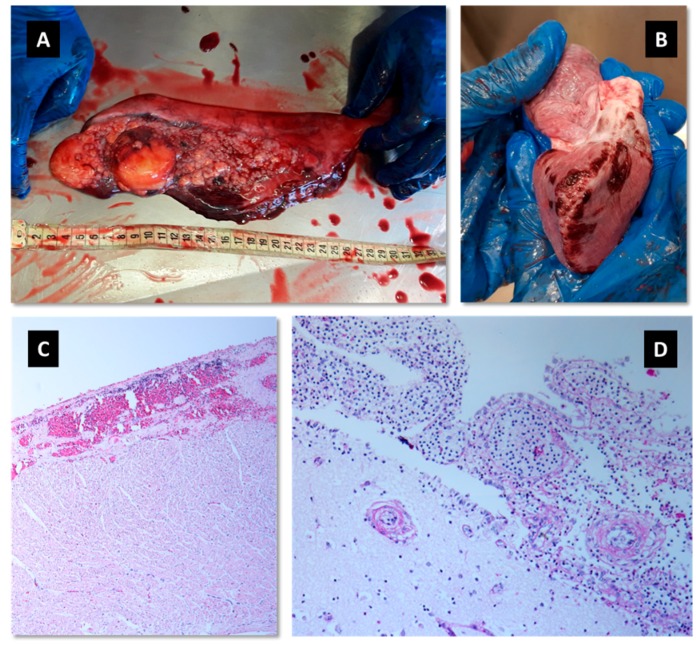
(**A**) Reproductive system: multifocal pyogranulomatos, irregular lumpy and firm texture, and extensive hemorrhages. (**B**) Heart: multifocal to coalescing epicardial hemorrhages and mild emphysema of the atrium. (**C**) Heart: reactive mesothel with hyperthrophy of the mesothelial cells resembling cuboidal epithelium, extensive hemorrhages and inflammation with mononuclear and polymorphonuclear cells. Inflammation is also present in the blood vessels’ wall (vasculitis) with some level of liquefying necrosis (hematoxylin and eosin, 100 magnification). (**D**) Brain: meningoencephalitis; fibrin and white blood cells in the blood vessels of the meninges. Perivascular cuffing with vascular liquefying necrosis (hematoxylin and eosin, 400 magnification).

The shark weighed 53 kg and its total length was 150 cm. At necropsy, no external signs of interaction with fishing nets or gear were observed. Gross pathological signs were recorded in the genital system (Figure 1A) and the heart (Figure 1B). 

Histopathology of the brain detected extensive diffuse meningoencephalitis, lymphocytes and macrophages. Polymorphonuclear cells were also present in a large number. Moreover, severe vasculitis was observed, with the area of liquefying necrosis of the blood vessel wall and perivascular cuffing in the neuropil (Figure 1D). Other histological findings observed in the heart were hemorrhages, granulomatous inflammation and a distinct hypertrophy of the mesothelial cells causing them to resemble cuboidal epithelium (Figure 1C). In the genital system, a multifocal to coalescing pyogranulomatous reaction with extensive mononuclear and polymorphonuclear inflammation, fibrin, corpora amylacea, and bacteria were present.

Initially, the bacteria was identified by matrix-assisted laser desorption ionization time-of-flight mass spectrometry (MALDI-TOF), which confirmed the presence of *S. agalactiae* in all isolates (the liver, spleen, kidney, uterine fluid and the brain).

The 16S rRNA sequencing confirmed 100% similarity to more than 30 *S. agalactiae* isolates, including strains isolated from Nile tilapia, *Oreochromis niloticus* (GenBank acc. no. KF111277.1), and pompano fish, *Trachinotus ovatus* (GenBank acc. no. KF826095.2).

According to the multiplex-PCR analysis, the *S. agalactiae* strain was found to be type Ia, bands sizes 521 and 1,826 bp, which were amplified and are indicative of the *cps*1aH gene (GenBank acc. no. AB028896). In addition, the band size 952 bp which was generated is indicative of the GBS-specific *dltS* gene (GenBank acc. no. AL766853) (data not shown). The WGS of the strain was analyzed for sequence type by gene extraction of the seven house-keeping genes (Table 1) and was typed as ST-7 (allelic profile 10-1-2-1-3-2-2). Recent publications link serotype Ia cases, specifically ST-7, with human infection, as well as with fish and water-related cases [14,15].

**Figure 2:**
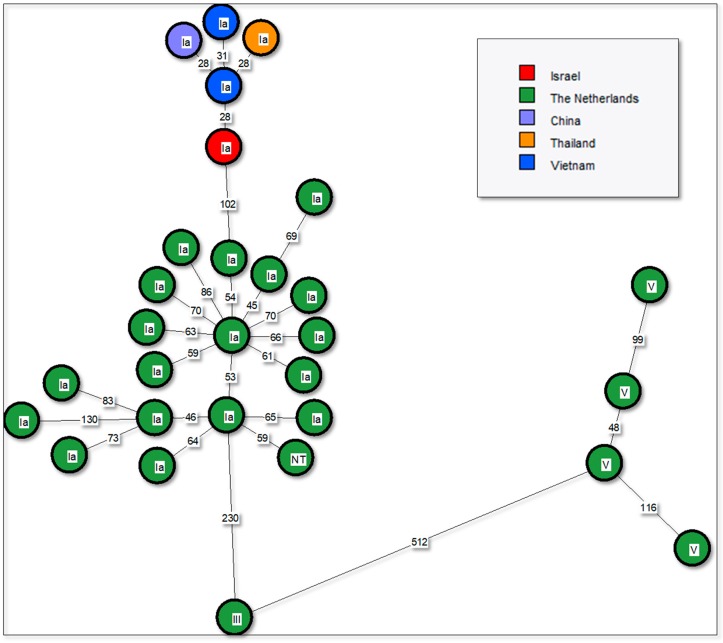
Whole genome MLST phylogenetic analysis of 29 publicly available global ST-7 sequences. Minimum spanning tree was based on wgMLST scheme (1,914 genes). The numbers on branches indicate allelic differences between strains. Group B *Streptococcus* (GBS) serotype is shown on top of the nodes. Color coding is by country of origin.

The phylogenetic analysis (Figure 2) indicates genetic clustering among global Ia-ST7. The Israeli strain was more similar to sequences from Asia and was distant from an Ia-ST7-cluster in The Netherlands.

## 4. Discussion

*Streptococcus agalactiae* is well known as a primary pathogen of both homoeothermic and poikilothermic organisms [11]. Most of the fish-streptococcosis caused by *S. agalactiae* were described in farmed species, particularly in tilapia [6], the second most cultured fish worldwide after carps [16]. *S*. *agalactiae* infections were also described in different wild fish species [7,8,9], however the number of publications is limited. 

Although the sandbar shark, *Carcharhinus plumbeus*, is one of the most common shark species in the Mediterranean Sea [1], the available knowledge regarding its health status and its susceptibility to pathogens are sparse. A bacterial infection that was reported in 1984 and described as *Vibrio damsela* and *V. carchariae* was associated with encephalitis in a naturally infected sandbar shark (also known as brown shark, *C. plumbeus*) which died in captivity at the National Aquarium of Baltimore, USA [17].

Our investigation revealed a bacterial multi-systemic infection caused by *S*. *agalactiae* in a female sandbar shark, *Carcharhinus plumbeus*, that was found moribund in July 2018 on Netanya's shoreline, Israel. Following post-mortem examination, it was clearly understood that the animal was sick. The heart and the genital system demonstrated a pathological appearance (Figure 1A,B). Histopathology confirmed pathological changes that were observed during the post-mortem examination (Figure 1B,C). The probable cause of death was a severe meningoencephalitis. Meningoencephalitis caused by *Carnobacterium maltaromaticum*-like bacteria was reported in wild stranded juvenile salmon sharks (*Lamna ditropis*) between 2002 and 2007 along approximately 300 miles of California’s shoreline, USA [18]. *C. maltaromaticum*-like bacteria is related to the *S*. *agalactiae* that was detected in our study as the *Carnobacteriaceae* family are in the same order of *Streptococcaceae* family (Lactobacillales).

The source of the infection remains unclear. Possible routes of infection and transmission for the *S. agalactiae* infection in wild sharks may include horizontal infection through waterborne exposure or oral transmission through ingestion of contaminated prey, as documented in relation to fish and other animals [8].

## 5. Conclusions

*Streptococcus agalactiae* are important pathogens of wild and cultured fish worldwide. Accurate reporting of the capsular genotype of *S. agalactiae* should be encouraged in scientific publications because many recent studies described *S. agalactiae* as a fish pathogen but fail to define the bacterial strain. Advanced molecular methods, as used in the current study, will allow for better comparison between studies and may speed up the scientific discovery of immune mechanisms, geographical distribution, inter-species infection patterns and potential biocontrol strategies.

More epidemiological studies are needed to provide insight into the likelihood and routes of inter-species transmission of strains that are associated with fish, marine mammals and humans. As *S*. *agalactiae* is a zoonotic agent, fishermen and researchers working with sharks should be aware of the transmission potential of the disease. This study broadens our knowledge regarding the health status of elasmobranches in this area and warrants further investigations of live and dead stranded elasmobranches for a better understanding of threats to endangered species.

## Figures and Tables

**Table 1:** Whole genome sequencing (WGS) of *Streptococcus agalactiae* available on pubMLST by wgMLST.

Year	Serotype	Country	Species	Reference
2015	Ia	China	Nile tilapia (*Oreochromis niloticus*)	4668 (WC1535)
2018	Ia	Israel	Sandbar shark (*Carcharhinus plumbeus*)	14,112 (ST33917)
2012	Ia	Thailand	Nile tilapia (*Oreochromis niloticus*)	4666 (JP9)
NA	Ia	The Netherlands	NA	3276 (ERR1624773)
NA	Ia	The Netherlands	NA	3280 (ERR1624777)
NA	Ia	The Netherlands	NA	3314 (ERR1624823)
NA	Ia	The Netherlands	NA	3319 (ERR1624830)
NA	Ia	The Netherlands	NA	3324 (ERR1624836)
NA	Ia	The Netherlands	NA	3327 (ERR1624843)
NA	Ia	The Netherlands	NA	3379 (ERR1624903)
NA	Ia	The Netherlands	NA	3455 (ERR1624990)
NA	V	The Netherlands	NA	3576 (ERR1625145)
NA	Ia	The Netherlands	NA	3584 (ERR1625155)
NA	Ia	The Netherlands	NA	3621 (ERR1625203)
NA	Ia	The Netherlands	NA	3631 (ERR1625214)
NA	Ia	The Netherlands	NA	3746 (ERR1625359)
NA	Ia	The Netherlands	NA	3754 (ERR1625374)
NA	V	The Netherlands	NA	3783 (ERR1625409)
NA	Ia	The Netherlands	NA	3804 (ERR1625443)
NA	V	The Netherlands	NA	3808 (ERR1625451)
NA	NT	The Netherlands	NA	3822 (ERR1625467)
NA	Ia	The Netherlands	NA	3837 (ERR1625486)
NA	Ia	The Netherlands	NA	3839 (ERR1625491)
NA	V	The Netherlands	NA	3957 (ERR1659816)
NA	III	The Netherlands	NA	4071 (ERR1672464)
NA	Ia	The Netherlands	NA	4216 (ERR1672631)
NA	Ia	The Netherlands	NA	4229 (ERR1672645)
2016	Ia	Vietnam	NA	4645 (SBVN)
2016	Ia	Vietnam	NA	4646 (3896VN)

NA, not available.

## Supplementary Materials

The following are available online at https://www.mdpi.com/2076-2615/10/2/284/s1, Video S1: Abnormal behavior of the wild sandbar shark, around 20 min before its death.
